# Combining systems pharmacology, metabolomics, and transcriptomics to reveal the mechanism of Salvia miltiorrhiza-Cortex moutan herb pair for the treatment of ischemic stroke

**DOI:** 10.3389/fphar.2024.1431692

**Published:** 2024-09-09

**Authors:** Chao Zhao, Xiaodan Bai, Yi Ding, Aidong Wen, Qiang Fu

**Affiliations:** ^1^ Department of Pharmaceutical Analysis, School of Pharmacy, Xi’an Jiaotong University, Xi’an, China; ^2^ Department of Pharmacy, Xijing Hospital, Air Force Medical University, Xi’an, China; ^3^ Precision Pharmacy and Drug Development Center, Department of Pharmacy, Tangdu Hospital, Air Force Medical University, Xi’an, China; ^4^ Department of Pharmaceutical Analysis, College of Pharmacy, Shenzhen Technology University, Shenzhen, China

**Keywords:** Salvia miltiorrhiza, cortex moutan, ischemic stroke, transcriptomics, metabolomics, VEGFA/PI3K/AKT pathway

## Abstract

Ischemic stroke (IS), predominantly triggered by blockages in cerebral blood flow, is increasingly recognized as a critical public health issue. The combination of Salvia miltiorrhiza (SM) and Cortex moutan (CM), traditional herbs in Eastern medicine, are frequently used for managing heart and brain vascular conditions. However, the exact mechanisms by which this herb pair (SC) combats IS remain largely unexplored. This investigation focuses on pinpointing the active constituents in SC that contribute to its protective role and deciphering the mechanisms countering cerebral ischemia, particularly in a middle cerebral artery occlusion (MCAO) rat model. We employed UPLC-Q-TOF-MS/MS alongside network pharmacology for predicting SC’s target actions against IS. Key ingredients were examined for their interaction with principal targets using molecular docking. The therapeutic impact was gauged through H&E, TUNEL, and Nissl staining, complemented by transcriptomic and metabolomic integration for mechanistic insights, with vital genes confirmed via western blot. UPLC-Q-TOF-MS/MS analysis revealed that the main components of SC included benzoylpaeoniflorin, salvianolic acid B, oxypaeoniflora, salvianolic acid A, and others. Network pharmacology analysis indicated that SC’s mechanism in treating IS primarily involves inflammation, angiogenesis, and cell apoptosis-related pathways, potentially through targets such as AKT1, TNF, PTGS2, MMP9, PIK3CA, and VEGFA. Molecular docking underscored strong affinities between these constituents and their targets. Our empirical studies indicated SC’s significant role in enhancing neuroprotection in IS, with transcriptomics suggesting the involvement of the VEGFA/PI3K/AKT pathway and metabolomics revealing improvements in various metabolic processes, including amino acids, glycerophospholipids, sphingomyelin, and fatty acids metabolisms.

## 1 Introduction

Ischemic stroke (IS) represents a critical neurological condition caused by insufficient cerebral blood supply, resulting in sensory and motor deficits, cognitive decline, and potentially fatal outcomes (Mendelson and Prabhakaran, 2021). It makes up approximately 85% of all strokes and stands as the third most significant contributor to worldwide mortality and disability, imposing a significant economic and public health load (Virani et al., 2020). Current IS treatments primarily involve t-PA thrombolytic therapy, but surgical thrombectomy can paradoxically worsen ischemia-reperfusion injury, exacerbating brain damage (Henderson et al., 2018). Therefore, the exploration of novel therapeutic strategies for stroke is imperative.

The *Salvia miltiorrhiza* Bunge (Salvia miltiorrhiza, SM) and *Paeonia × suffruticosa* Andrews (Cortex moutan, CM) herb pair (SC) is a traditional combination in Eastern medicine known for its efficacy for its blood-flow enhancing properties. It has been historically applied to a spectrum of ailments, including cardiovascular and cerebrovascular diseases, liver diseases, pneumonia, renal diseases, and even cancers (Sun et al., 2005; Lee et al., 2006; Yang et al., 2010; Li et al., 2022; Zhang M. et al., 2022). SM contains bioactive compounds such as tanshinones and phenolic acids, known for their diverse pharmacological effects, including antioxidant, endothelial protective, anti-atherosclerotic, anti-inflammatory, and vasodilatory properties. For instance, salvianolate has been verified to protect against cerebral ischemic injury in diabetic rats by regulating inflammatory factors and the antioxidant pathway (Wang et al., 2017). Additionally, SM ethanol extract can alleviate cerebral ischemic injury in rats, inhibit thrombosis formation, and attenuate platelet aggregation (Fei et al., 2017). CM, on the other hand, contains paeonol, a prominent component with potent vasodilatory effects, potentially reducing intracellular calcium concentration by modulating Ca^2+^ influx and release (Li et al., 2010). However, due to the complexity and cooperativity of SC, the molecular mechanisms underlying its neuroprotective effects remain elusive.

The Phosphoinositide 3-Kinase (PI3K)/AKT pathway, crucial in cell survival and metabolism, plays a pivotal role in IS pathology (Liu et al., 2023b). Hypoxic conditions and inflammatory cytokines can stimulate the release of growth factors, many of which activate the AKT signal transduction pathway (Koh and Lo, 2015). These growth factors promote neuroprotection by activating AKT and influencing processes such as inflammation, apoptosis, and angiogenesis. Gomisin N, isolated from *Schisandra chinensis* (Turcz.) Baill, has been shown to attenuate IS-induced neuroinflammation and autophagy by regulating the PI3K/AKT/mTOR signaling pathway (Li R. et al., 2023). Recent research suggests that PI3K/AKT signaling can modulate arterial thrombosis (Liberale et al., 2023) and attenuate blood-brain barrier disruption in the penumbra, consequently improving post-ischemia neurological function. Vascular endothelial growth factor (VEGF) is a well-known angiogenic factor, that exerts its angiogenesis promotion and neuroprotective effects by binding to the VEGF receptor. SM has been demonstrated to promote angiogenesis in ischemic heart disease (Li X. et al., 2023).

The aforementioned findings suggest that SC may have therapeutic potential for IS; however, no relevant reports have been published to date. In this research, we aim to verify the neuroprotective effects of SC via the VEGFA/PI3K/AKT signaling pathway. Firstly, we will identify the components of SC and establish MCAO rat models to explore the effects of SC on neuroprotective development. Additionally, we will employ a multidisciplinary approach, including systems pharmacology, transcriptomics, and metabolomics, to unravel the mechanisms through which SC acts against cerebral ischemic injury.

## 2 Materials and methods

### 2.1 Preparation of the SC herb pair decoction

Salvia miltiorrhiza (SM) and Cortex moutan (CM) were procured from Xijing Hospital (Xi’an, China). SC herb pair decoction was prepared according to the method of the Chinese Pharmacopoeia (2020 Edition). First, Cortex moutan (100 g) was powdered and extracted by adding six times the volume of water for distillation. Then, Salvia miltiorrhiza (200 g) and the dregs of Cortex moutan were extracted by adding six times the volume of water boiled twice for 2 h, filtered, mixed, and then combined with the Cortex moutan distillate. The resulting aqueous extract was concentrated to 2 g/mL for Salvia miltiorrhiza and 1 g/mL for Cortex moutan crude drug.

### 2.2 Determination of SC components

The SC concoction underwent vacuum freeze-drying and was then analyzed through ultra-high-performance liquid chromatography-quadrupole time-of-flight tandem mass spectrometry (UPLC-Q-TOF-MS/MS, Waters Acquity I-CLASSTM UPLC, Waters Xevo G2-XS, United States). The SC powder was dissolved in 30% methanol (2 mg/mL), agitated via vortex, and centrifuged at 14,000 rpm for 10 min. The supernatant was then filtered through a 0.22-μm filter for analysis. An ACQUITY HSS T3 column (2.1 × 100 mm, 1.8 μm, United States) at 40°C with a 0.4 mL/min flow rate was used for chromatographic separation. The gradient elution program utilized a mobile phase containing acetonitrile (A) and 0.1% formic acid water (B). The program was as follows: 0–2 min, 95% B; 2–14 min, 90%–65% B; 14–15 min, 65%–60% B; 15–25 min, 60%–20% B; 25–25.1 min, 20%–95% B; 25.1–30 min, 95% B. Mass spectrometry was performed in both negative and positive modes, with data spanning m/z 50–1,500 Da. Analysis was facilitated by Waters UNIFI software (version 1.9.1).

### 2.3 Experimental design and animal treatments

Sixty adult male Sprague-Dawley rats (250 ± 20 g) were obtained from the Air Force Military Medical University’s Experimental Animal Center (Xi’an, China, license number for animal production: SCXK-2019-001). Animals were housed under a 12-h light-dark cycle at 23°C ± 2°C and 50%–70% humidity, following approval by the Institutional Animal Care and Utilization Committee (IACUC-202305177). The rats were divided into six groups for various treatments (n = 10): sham, MCAO, MCAO + SC-L (3.75 g/kg/day), MCAO + SC-M (7.5 g/kg/day), MCAO + SC-H (15 g/kg/day), and MCAO + ginaton (0.1 g/kg/day). The drug administration of SC and ginaton groups was conducted through oral gavage from 3 days pro-surgery to 14 days post-surgery. Sham and MCAO groups received saline.

### 2.4 MCAO/R model establishment

The middle cerebral artery occlusion/reperfusion (MCAO/R) model was induced as per established methods (Bai et al., 2023). The external carotid artery was exposed through a midline incision after positioning the rats on a warming platform following their anesthesia with pentobarbital sodium (30 mg/kg). A nylon filament was used to occlude the middle cerebral artery for 2 h, followed by reperfusion. The sham procedure was identical, excluding filament insertion.

### 2.5 Neurological and infarct volume assessment

Fourteen days after MCAO/R, neurological function was assessed using the Zea longa 5-grade scoring system (Longa et al., 1989; Liu et al., 2023a). Brain slices were stained with 2,3,5-triphenyl tetrazolium chloride (TTC) for infarct volume determination, and analyzed with Image-Pro Plus software.

### 2.6 Rotarod test and corner test

The rotarod test (LE8200 Panlab, Harvard Apparatus, United States) was conducted to evaluate the coordination and balance in the forelimbs and hindlimbs. The rats were subjected to the accelerating rotor mode, which varied from 5 to 40 rpm over a duration of 300 s. An investigator, blinded to the experimental groups, recorded the duration each animal stayed on the rod. For statistical analysis, the final score was represented as the average time a rat was able to maintain its position on the rod across two trials (Xia et al., 2023). The corner test serves as a commonly utilized functional evaluation for unilateral sensorimotor cortical impairment. In a home cage, two cardboard plates were positioned at a 30° angle. Each rat was positioned between these plates and permitted to navigate freely to the corner. Following the ischemic and reperfusion injury to the sensorimotor cortex, the rats exhibited a tendency to turn biasedly towards the side corresponding with their brain damage (Han et al., 2015). The frequency of left turns made by the rats during 10 trials was documented.

### 2.7 H&E, TUNEL, and Nissl staining

Histological analysis was conducted to assess histological damage, neuronal loss, and apoptosis in the ischemic hemisphere. Brain tissue underwent fixation, paraffin embedding, and sectioning for histological analyses. To evaluate neuronal apoptosis levels, the stained tissue sections were observed under a fluorescence microscope at a magnification of 200x to visualize TUNEL-positive cells. Additionally, observations of H&E staining and Nissl staining were made at a magnification of 400x. This allowed for the assessment of various aspects of the brain tissue.

### 2.8 Network pharmacology analyses

SC’s active components, identified via UPLC-Q-TOF-MS/MS, were assessed for Oral Bioavailability (OB) and Drug Similarity (DL) using the Traditional Chinese Medicine Systems Pharmacology (TCMSP) database. Only compounds with OB ≥ 30% and DL ≥ 0.18 were identified and preselected as active ingredients. Target predictions were made using Swiss Target Prediction, while ischemic stroke-related targets were collated from multiple databases, including Online Mendelian Inheritance in Man (OMIM), Genecards, DrugBank, and Therapeutic Target Database (TTD). Overlapping targets underwent protein-protein interaction (PPI) analysis using STRING, Gene Ontology (GO) and Kyoto Encyclopedia of Genes and Genomes (KEGG) pathway enrichment analyses were conducted through DAVID, and KEGG results were visualized using the bioinformatics platform (http://www.bioinformatics.com.cn/). The final composite-target-pathway network was created using Cytoscape software (version 3.7.1).

### 2.9 Molecular docking analyses

Molecular structures of SC components were sourced from PubChem and prepared Mol2 files using Chem3D. Protein targets’ crystal structures with high resolution were retrieved from the RCSB Protein Data Bank (PDB), and edited using PyMOL (version 1.7.2). The optimal conformations of ingredients at the active sites of protein targets were predicted using Auto Dock Tools (version 1.5.7). The interaction and binding modes with the lowest binding energies were analyzed using Discovery Studio 2020 Client and visualized.

### 2.10 Molecular dynamics simulation

Based on the promising outcomes achieved from molecule docking, additional insights into the dynamic behavior and binding mechanisms were revealed through molecular dynamics simulation (MD). The MD simulations were performed using Gromacs 2022 software, where small molecules were modeled with the GAFF force field, proteins with the AMBER14SB force field, and a TIP3P water model was employed to create a simulation environment for the complexes. These simulations followed periodic boundary conditions under constant pressure and temperature. To maintain stability during MD simulations, all hydrogen bonds were constrained using the LINCS algorithm with a time step of 2 fs Electrostatic interactions were computed utilizing the Particle-Mesh Ewald (PME) method within a cutoff distance of 1.2 nm. Non-bonded interactions had a cutoff set at 10 Å and updated every 10 steps. Temperature was kept at 298 K using V-rescale method for temperature coupling while pressure was regulated at 1 bar via Berendsen technique. Equilibrium simulations including both NVT and NPT ensembles were conducted for 100 ps at 298 K, followed by an extensive MD simulation of the complex for 100 ns with conformational snapshots recorded every 10 ps Post-simulation analysis involved trajectory examination using Visual Molecular Dynamics (VMD) and PyMOL software tools, along with calculation of root mean square deviation (RMSD) and root mean square fluctuation (RMSF) between protein and small molecule ligands employing g_mmpbsa program.

### 2.11 Transcriptomic analyses

Transcriptome analysis was performed on ischemic penumbra brain tissues from the sham, MCAO, and SC-H groups (n = 3). The extraction of Total RNA was carried out utilizing the TRIzol reagent, followed by assessing its purity and integrity. The reverse transcription of mRNA resulted in the creation of cDNA, and subsequently, the construction of cDNA libraries commenced. Afterward, sequencing using the 2 × 150 bp paired-end method was performed on an Illumina Novaseq™ 6000 sequence platform (LC-Bio Technology Co., Ltd. Hangzhou, China). The transcriptome was aligned to the reference genome using HISAT2 (version 2.2.1), and differential expression analysis was performed using the DEGseqR package with a significance threshold (q-value ≤0.05, |log2FC|>1). GO enrichment and KEGG pathway analysis were performed to identify significantly enriched GO terms and pathways related to the effects of SC on ischemic stroke based on differentially expressed genes (DEGs).

### 2.12 Metabolomics analyses

Serum samples from the sham, MCAO, and SC-H groups were subjected to extraction using 50% methanol buffer and subsequent centrifugation. Ultra-high-performance liquid chromatography (ThermoFisher, Germany) coupled with a high-resolution tandem mass spectrometer TripleTOF6600plus (SCIEX, United States) was used for analysis (LC-Bio Technology Co., Ltd. Hangzhou, China). Mass spectrometry data were processed using XCMS software, and differential metabolites were identified based on variable importance in the projection (VIP) scores >1 and q-value ≤0.05. Differential metabolites were identified and analyzed for pathway implications using KEGG and HMDB databases.

### 2.13 Multi-omics integrated analysis

To investigate the relationship among various omics data, an integrated analysis was performed. Initially, homologous gene conversion was executed, converting all IDs to human gene equivalents, followed by standardization through the UniProt database. Subsequently, the predicted targets of SC were compared with IS-related genes and differentially expressed genes. The overlapping targets were deemed as high-confidence candidates and were analyzed utilizing the DAVID database. The KEGG outcomes were illustrated using a bioinformatics platform (http://www.bioinformatics.com.cn/). Ultimately, the Joint Pathway Analysis module in MetaboAnalyst 6.0 synthesized results from transcriptomic and metabolomic analyses for the identified treatment-related DEGs and differential metabolites. Scatterplots were generated to illustrate the identified KEGG pathways, with those exhibiting a *p*-value of less than 0.05 considered significantly enriched.

### 2.14 Western blotting

Standard western blotting was performed following the established methodology (Zhang et al., 2021). The brain tissue samples were homogenized and lysed before undergoing centrifugation. Protein concentrations were ascertained using a bicinchoninic acid assay. Proteins were then separated via SDS-PAGE and transferred onto PVDF membranes. To reduce non-specific interactions, membranes were blocked with 5% skim milk. Overnight incubation at 4°C with primary antibodies against PI3K, p-PI3K, AKT, p-AKT, HIF-1α, β-Actin, GAPDH (1:1,000, CST), VEGFA (1:1,000, Abcam), and MMP9 (1:1,000, Service Bio) was performed. This was followed by incubation with horseradish peroxidase-conjugated secondary antibody (goat anti-rabbit IgG, 1:3,000, Service Bio) and visualization. Protein band detection and quantification were conducted using ImageJ software (version 1.53).

### 2.15 Immunofluorescence assay

In the immunofluorescence assay, selected brain tissue sections underwent staining with primary antibodies against rabbit anti-VEGFA and anti- HIF-1α (1:500, CST). After appropriate blocking and washing, sections were incubated with fluorescently labeled secondary antibodies. Nuclei were counterstained with DAPI, and the slides were examined under a Nikon Eclipse C1 microscope, providing insights into the cellular and molecular changes induced by SC.

### 2.16 Statistical analysis

Data presentation utilized mean ± SD format, with group differences assessed through one-way ANOVA and Tukey’s *post hoc* test. A *p*-value <0.05 was deemed statistically significant. GraphPad Prism software (version 10.0.2) facilitated all statistical analyses.

## 3 Results

### 3.1 Chemical compounds analysis of SC herb pair decoction

**FIGURE 1:**
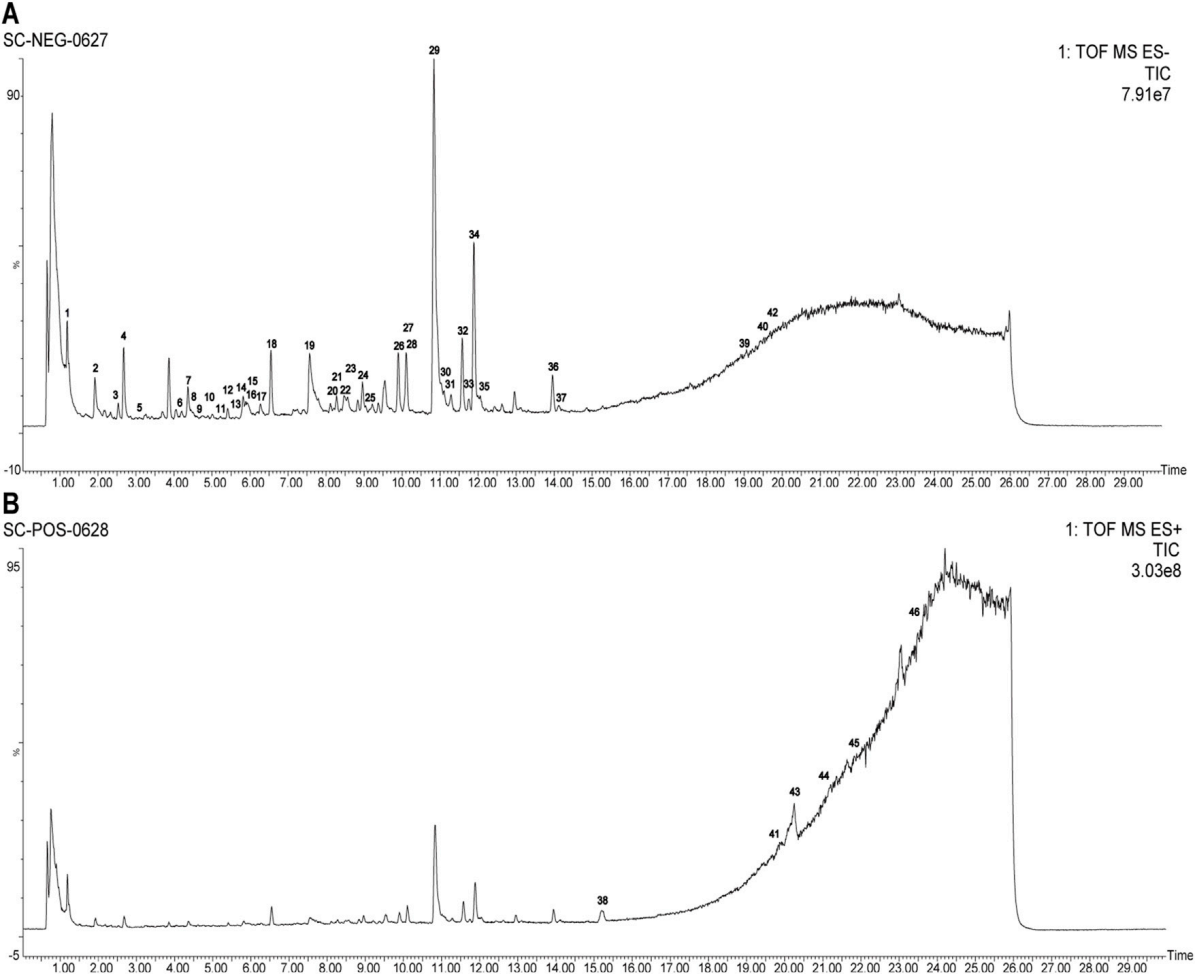
Comprehensive LC-MS/MS profiling of SC herb pair constituents. **(A)** Total ion chromatogram displaying the profile of SC herb pair in negative ion mode. **(B)** Total ion chromatogram displaying the profile of SC herb pair in positive ion mode.

Employing UPLC-Q-TOF-MS/MS, 46 principal compounds were identified in SC herb pair decoction, including phenolic acids, tanshinones, paeoniflorins, paeonol, and tannins, based on retention times and MS spectra from literature or reference standards (Figure 1; Table 1). These compounds were recognized as potentially active and used for subsequent target and pathway prediction via network pharmacology.

**TABLE 1:** Forty-six key chemical constituents were identified from the SC herb pair using UPLC-Q-TOF-MS/MS in the positive and negative ion modes.

No.	Component name	RT (min)	Neutral mass (Da)	Observed m/z	Mass error (ppm)	Formula	Adducts	MS Fragments	Plant material
1	Mudanoside B	1.25	464.1166	463.1082	−2.5	C_18_H_24_O_14_	−H	169.0126,236.0559, 281.0875,403.0855	CM
2	Gallic acid	1.93	170.0215	169.014	−1.2	C_7_H_6_O_5_	−H	107.0135,123.0083,125.0243	CM
3	4-Paeoniflorinsulfonic acid	2.54	560.12	559.1145	3.1	C_23_H_28_O_14_S	−H	137.0243,179.0348,213.0221,259.0280,319.0131,375.0749,421.0811	CM
4	Danshensu	2.68	198.0528	197.0457	0.7	C_9_H_10_O_5_	−H	109.0290,123.0452,135.0452,153.0549,179.0351	SM
5	Protocatechuic acid	3.11	154.0266	153.019	−2.4	C_7_H_6_O_4_	−H	109.0292	SM
6	Protocatechualdehyde	4.2	138.0317	137.0246	1.2	C_7_H_6_O_3_	−H	92.0265,108.0216	SM
7	Oxypaeoniflora	4.37	496.1581	495.1529	4.2	C_23_H_28_O_12_	−H	93.0348,137.0245,165.0558,333.0982,465.1413	CM
8	4-Hydroxybenzoic acid	4.43	138.0317	137.0245	0.6	C_7_H_6_O_3_	−H	93.0346	CM
9	(−)-Catechin	4.51	290.079	289.0726	3	C_15_H_14_O_6_	−H	125.0240,135.0450,137.0243,179.0351	CM
10	Albiflorin	4.9	480.1632	525.1635	4.1	C_23_H_28_O_11_	+HCOO	167.0348,177.0546,363.1078	CM
11	Caffeic acid	5.23	180.0423	179.0351	0.7	C_9_H_8_O_4_	−H	135.0448	SM
12	Paeonolide	5.41	460.1581	505.1583	4	C_20_H_28_O_12_	+HCOO	122.0368,150.0322,165.0560,197.0454	CM
13	(−)-Epicatechin	5.67	290.079	289.0722	1.4	C_15_H_14_O_6_	−H	137.0241	CM
14	Apiopaeonoside	5.82	460.1581	505.1581	3.6	C_20_H_28_O_12_	+HCOO	122.0368,135.0450,149.0451,165.0560,233.0665,293.0880	CM
15	Paeoniflorinsulfonic acid	5.97	544.1251	543.1188	1.8	C_23_H_28_O_13_S	−H	165.0553,259.0280,339.0509,381.0651,421.0231	CM
16	Paeonoside	5.97	328.1158	373.1145	1.2	C_15_H_20_O_8_	+HCOO	125.0240,150.0316,165.0555	CM
17	Galloyloxypaeoniflorin	6.27	648.169	647.1648	4.7	C_30_H_32_O_16_	−H	169.0140,271.0462,313.0567,491.1197,629.1516	CM
18	Paeoniflorin	6.54	480.1632	525.1613	0.1	C_23_H_28_O_11_	+HCOO	121.0295,165.0560,327.1097,357.1190,449.1469	CM
19	Suffruticoside C	7.71	612.169	611.1642	4	C_27_H_32_O_16_	−H	125.0242,165.0556,169.0145,417.0868	CM
20	Suffruticoside A	8.21	612.169	611.1639	3.4	C_27_H_32_O_16_	−H	125.0238,165.0554,289.0712,445.0989	CM
21	Galloylpaeoniflorin	8.28	632.1741	631.1697	4.6	C_30_H_32_O_15_	−H	125.0240,169.0143,271.0466,313.0571,491.1199,509.1305,613.1586	CM
22	Mudanpioside E	8.37	526.1686	525.16	−2.5	C_24_H_30_O_13_	−H	135.0449,165.0555,197.0451,449.1447	CM
23	Pentagalloylglucose	8.49	940.1182	939.1165	5.9	C_41_H_32_O_26_	−H	125.0243,169.0144,431.0624,599.0678,617.0783,769.0922	CM
24	Salvianolic Acid D	8.96	418.09	417.0833	1.5	C_20_H_18_O_10_	−H	135.0451,157.0293,175.0400,179.0351,197.0456,295.0616,321.0408	SM
25	Mudanpioside H	9.05	616.1792	615.1741	3.5	C_30_H_32_O_14_	−H	137.0243,281.0668,477.1397,495.1503	CM
26	Rosmarinic Acid	9.90	360.0845	359.0778	1.7	C_18_H_16_O_8_	−H, +Cl	135.0453,161.0245,179.0353,197.0460,219.0274	SM
27	Benzoylpaeoniflorin sulfonate	10.06	648.1513	647.1469	4.4	C_30_H_32_O_14_S	−H	121.0292,213.0218,259.0282,479.1001,525.1073	CM
28	Lithospermic Acid	10.12	538.1111	537.1056	3.3	C_27_H_22_O_12_	−H	109.0296,135.0452,185.0246,197.0458,203.0352,295.0618,313.0721,493.1153	SM
29	Salvianolic Acid B	10.84	718.1534	717.1491	4.1	C_36_H_30_O_16_	−H	135.0453,185.0247,295.0619,321.0401,339.0514,457.0932,493.1154,519.0943	SM
30	Mudanpioside C	11.11	600.1843	599.1795	4.1	C_30_H_32_O_13_	−H	121.0292,137.0244,177.0556,281.0671,431.1350,477.1402	CM
31	Salvianolic acid E	11.29	718.1534	717.1491	4.2	C_36_H_30_O_16_	−H	109.0295,135.0449,185.0240,295.0619,321.0415,339.0519,519.0951	SM
32	Salvianolic acid L	11.59	718.1534	717.1494	4.7	C_36_H_30_O_16_	−H	109.0295,135.0449,185.0245,295.0620,321.0412,339.0520,519.0949	SM
33	Benzoyloyxpaeoniflorin	11.76	600.1843	599.1797	4.5	C_30_H_32_O_13_	−H	137.0245,195.0659,333.0981,449.1442,477.1405	CM
34	Salvianolic Acid A	11.89	494.1213	493.1151	2.1	C_26_H_22_O_10_	−H	109.0296,135.0454,185.0245,203.0355,269.0819,295.0617,313.0725,335.0545	SM
35	Methyl Rosmarinate	12.06	374.1002	373.0936	2	C_19_H_18_O_8_	−H	135.0453,179.0350,197.0456,325.0722,343.0825	SM
36	Benzoylpaeoniflorin	13.96	584.1894	629.19	3.9	C_30_H_32_O_12_	+HCOO	121.0295,165.0557,413.1238,431.1355,553.1734	CM
37	Salvianolic Acid C	14.12	492.1057	491.0995	2.4	C_26_H_20_O_10_	−H	135.0449,179.0346,197.0451,265.0501,293.0460,311.0559,473.0878	SM
38	Paeonol	15.20	166.063	167.0697	−3.7	C_9_H_10_O_3_	+H	91.0553,121.0641	CM
39	Tanshinone Ⅵ	19.08	296.1049	295.0974	−0.5	C_18_H_16_O_4_	−H	222.0675,237.0912,249.0932,265.0858	SM
40	Tormentic Acid	19.44	488.3502	487.3428	−0.2	C_30_H_48_O_5_	−H	235.1340,443.3533,469.3322	SM
41	Isocryptotanshinone	19.61	296.1412	297.1477	−2.6	C_19_H_20_O_3_	+H	141.0692,157.0998,222.0666	SM
42	Salviolone	19.63	268.1463	313.1451	1.9	C_18_H_20_O_2_	+HCOO	198.1055,213.1278,226.1001	SM
43	Dihydrotanshinone I	20.14	278.0943	279.1009	−2.4	C_18_H_14_O_3_	+H	141.0696,169.0636	SM
44	Epidanshenspiroketallactone	21.21	268.1099	269.1161	−4.3	C_17_H_16_O_3_	+H	127.0541,129.0678,141.0691,143.0839	SM
45	Cryptotanshinone	21.82	296.1412	297.1475	−3.4	C_19_H_20_O_3_	+H	141.0691,235.0731,254.0923,267.0999	SM
46	Tanshinone IIA	23.54	294.1256	295.1308	−4.9	C_19_H_18_O_3_	+H	184.3843,283.2235	SM

### 3.2 SC treatment alleviates MCAO-associated injury in rats

**FIGURE 2:**
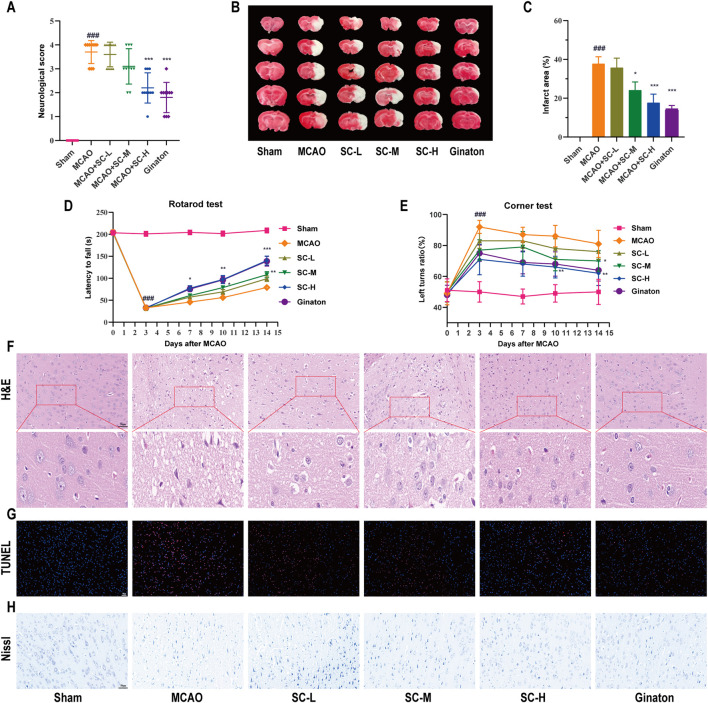
Therapeutic efficacy of SC herb pair in mitigating ischemic stroke induced by MCAO (n = 10). **(A)** Graphical representation of neurological deficit scores among different rat groups. **(B)** TTC staining images depicting cerebral infarction in various treatment groups. **(C)** Quantitative analysis of cerebral infarct area across different groups. **(D)** Latency time for rats to fall from the rotating rod. **(E)** The turning bias in the corner test. **(F–H)** Microscopic images of brain tissues stained with H&E, TUNEL, and Nissl (Scale bar = 50 μm). *Statistical significance indicated: *p < 0.05, **p < 0.01, ***p < 0.001 vs MCAO group; #*p* < 0.05, ##*p* < 0.01, ###*p* < 0.001 vs sham group.

SC treatment markedly improved neurological outcomes in MCAO-induced rats, significantly reducing deficit scores (*p* < 0.01) (Figure 2A). Infarct volumes, assessed by TTC staining, were notably less in SC-H and ginaton groups (*p* < 0.001) (Figures 2B, C). Next, we performed the rotarod test and corner test to assess motor function and found that the SC-treated rats significantly increased latency to fall off the rod (Figure 2D), and increased the number of left turns in the corner test (Figure 2E). H&E staining demonstrated that the MCAO group exhibited nuclear deformation and neuronal loss, leading to serious cell structure damage in the penumbra, while reduced cellular damage in SC-treated groups compared to the MCAO group (Figure 2F). TUNEL staining (Figure 2G) showed a dose-dependent reduction in apoptosis in the SC-treated groups. Nissl staining (Figure 2H) revealed a significant reduction in the number of Nissl bodies and disrupted neuronal morphology in the MCAO group, while SC-treated groups exhibited an increase in Nissl bodies and improved neuronal morphology. These findings demonstrate that SC reduces brain damage, enhances neuroprotection, and restores brain function after ischemic stroke.

### 3.3 Predictive network pharmacology

**FIGURE 3:**
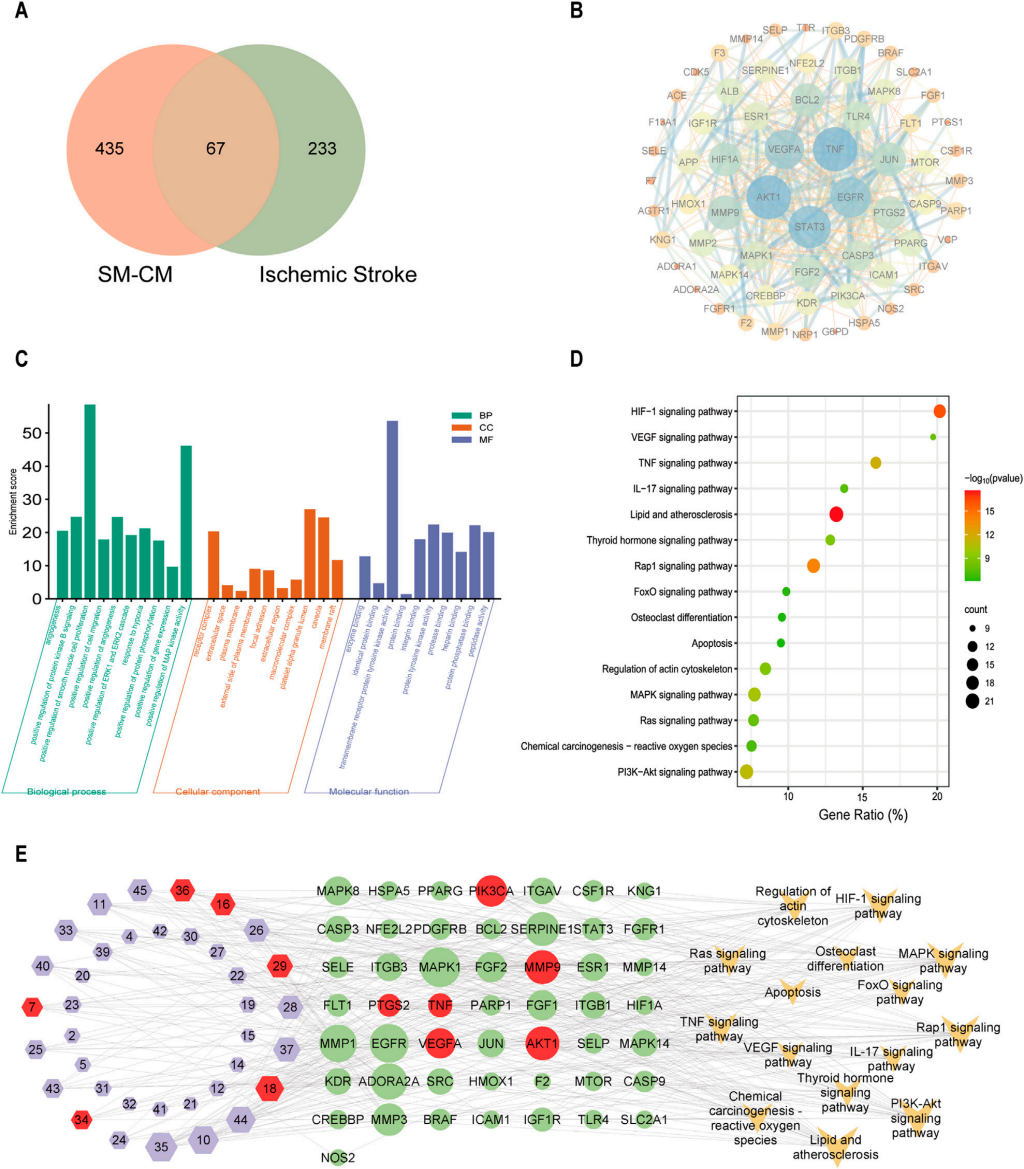
Network pharmacology analysis elucidating SC’s mechanism of action in ischemic stroke. **(A)** Venn diagram depicting the overlap of targets between SC and ischemic stroke. **(B)** PPI network of shared targets. **(C)** GO enrichment analysis results of shared targets. **(D)** KEGG pathway analysis of core genes in the network. **(E)** Integrated network connecting ischemic stroke targets with SC ingredients.

A total of 502 predicted targets for effective active compounds of SC were identified by using the Swiss Target Prediction, and 300 targets with high relevance scores were filtered from 4824 IS-related targets obtained in GeneCards (4,789), OMIM (20), TTD (17), and Drugbank (57) database using the keyword “Ischemic stroke” on 30 April 2023. Ultimately, 67 overlapping targets between SC and IS were identified as pivotal targets (Figure 3A). The PPI network highlighted important proteins including AKT1, TNF, PTGS2, MMP9, PIK3CA, VEGFA, and so on (Figure 3B). GO and KEGG pathway analyses connected these targets to positive regulation of angiogenesis, response to hypoxia, apoptosis regulation, and pathways like PI3K-AKT, TNF, VEGF, and HIF-1α signaling in IS (Figures 3C, D). A comprehensive network illustrating the interactions among SC components, targets, and pathways was constructed (Figure 3E).

### 3.4 Molecular docking and molecular dynamics simulation of key ingredients

**FIGURE 4:**
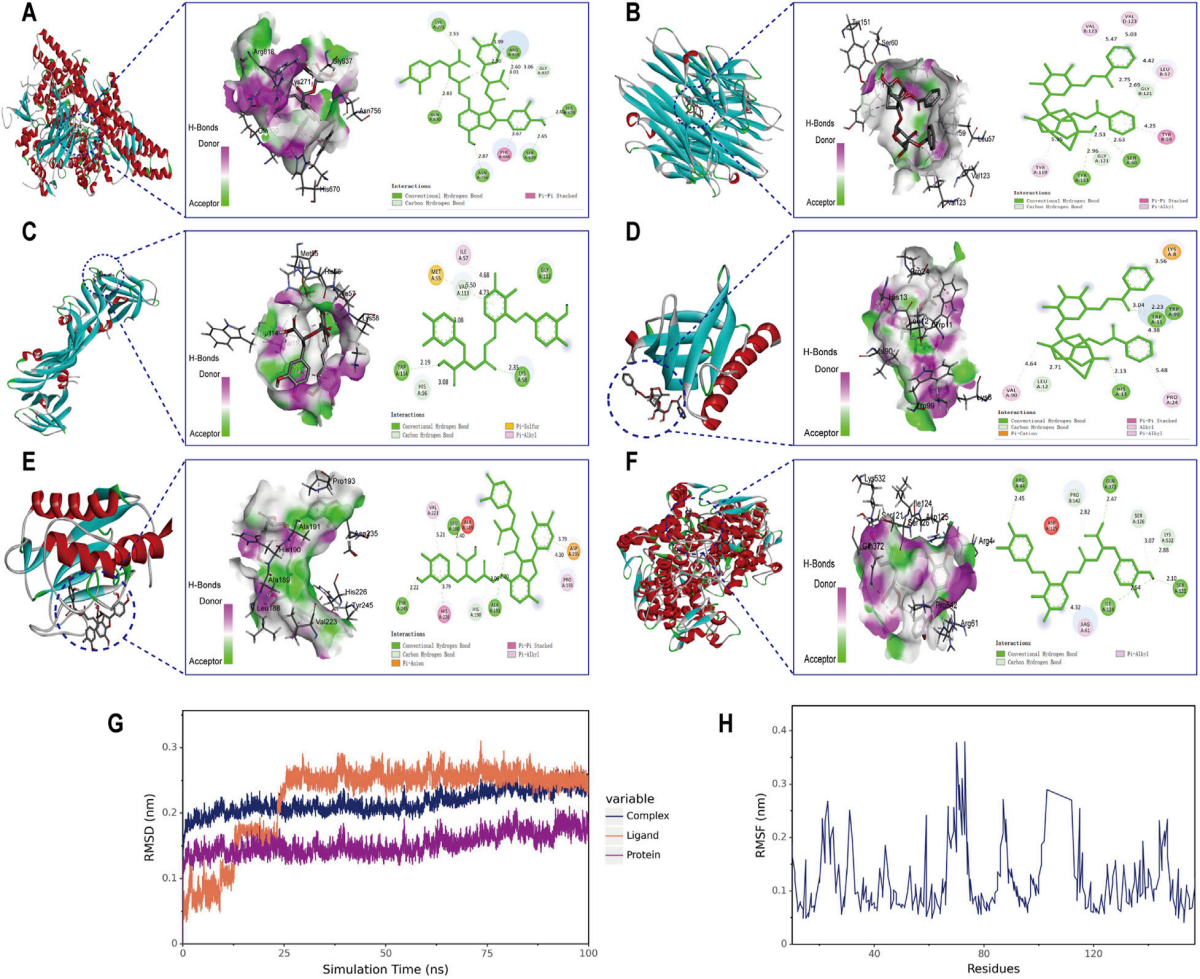
Molecular docking interaction diagrams and molecular dynamics simulation between SC components and target proteins. Schematic diagrams (3D and 2D) of the docking models. **(A)** Salvianolic acid B with PIK3CA, **(B)** benzoylpaeoniflorin with TNF **(C)** salvianolic acid A with VEGFA, **(D)** benzoylpaeoniflorin with AKT1 **(E)** salvianolic acid B with MMP9, and **(F)** salvianolic acid B with PTGS2. **(G)** RMSD plot during molecular dynamic simulations of salvianolic acid B with PIK3CA. **(H)** RMSF plot during molecular dynamic simulations of salvianolic acid B with PIK3CA.

Molecular docking analysis was conducted between six target proteins identified in the PPI analysis and six potentially active compounds. The results revealed good binding potential between key components in SC and target genes, including PIK3CA, TNF, VEGFA, AKT1, MMP9, and PTGS2 (Figures 4A–F). A total of 31 pairs were highlighted with significant binding activity with the affinity < −7 kcal/mol (Table 2). Next, salvianolic acid B, benzoylpaeoniflorin, and salvianolic Acid A were selected as core components for molecular dynamics simulations with PIK3CA, TNF, VEGFA, AKT1, MMP9, and PTGS2. The binding of salvianolic acid B to the ligands reached a steady state rapidly without excessive fluctuations. After a brief fluctuation, the binding of benzoylpaeoniflorin and salvianolic acid A to the ligand also formed a steady state. The results are shown in Figures 4G, H and Supplementary Figure S1.

**TABLE 2:** The docking scores and binding sites of key ingredients with six target proteins.

Target	Affinity (kcal/mol)					
	Paeonoside	Paeoniflorin	Salvianolic acid A	Salvianolic acid B	Benzoylpaeoniflorin	Oxypaeoniflora
PIK3CA(4JPS)	−7.1	−9.3	−8.8	−10.0	−9.0	−8.9
TNF(2AZ5)	−7.1	−8.3	−8.2	8.6	8.7	8.4
VEGFA (4QAF)	−6.0	−7.3	−8.3	7.9	8.1	8.1
AKT1(1H10)	−5.3	−6.6	−7.0	7.0	7.3	6.3
MMP9(4XCT)	−6.4	−8.3	−8.8	−9.9	−8.1	−8.7
PTGS2(5F19)	−7.0	−8.5	−8.8	9.8	8.8	9.4

### 3.5 Transcriptomic insights

**FIGURE 5:**
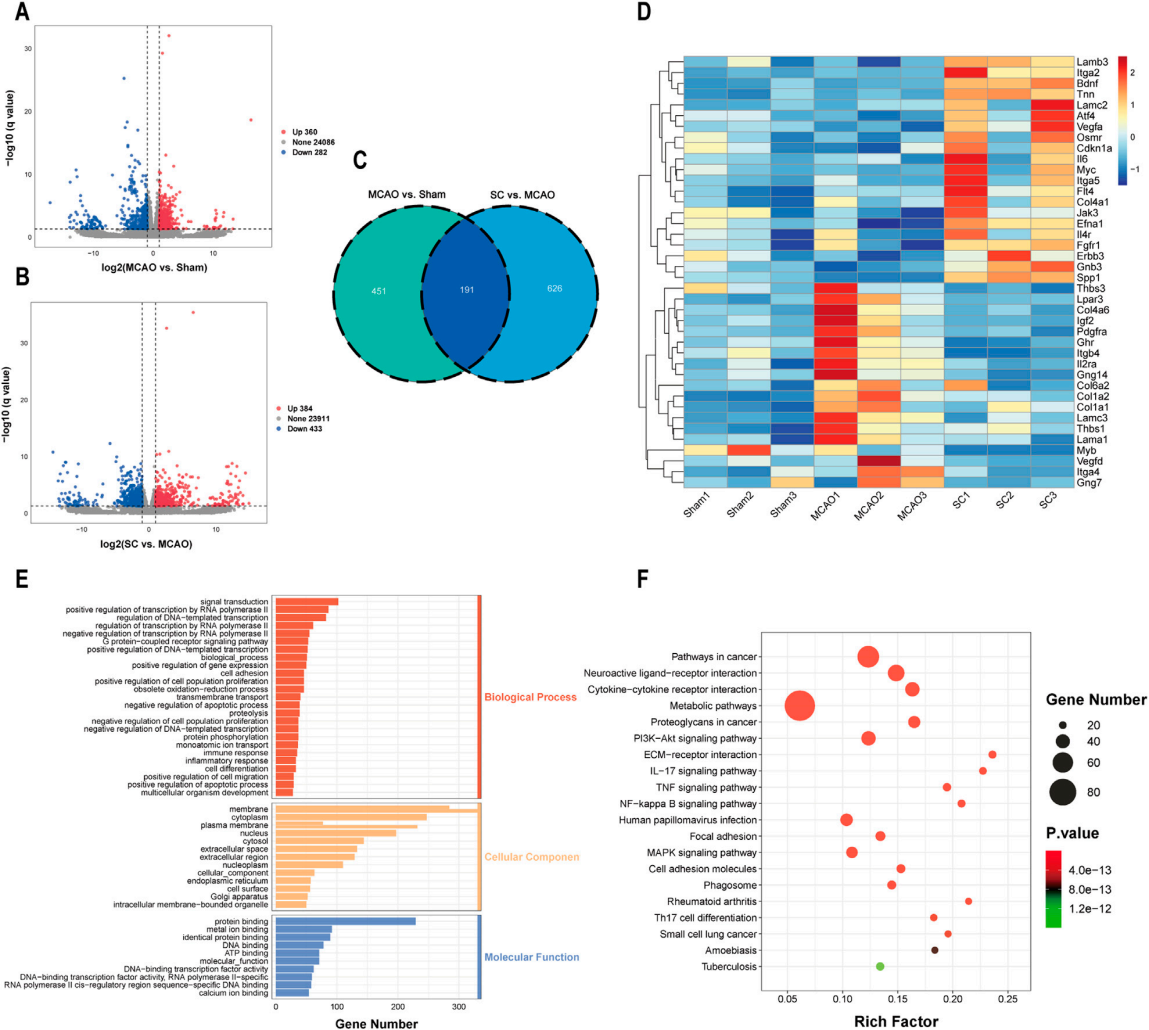
Transcriptomic revealed DEGs for the anti-ischemic stroke effect of SC (n = 3). **(A, B)** Volcano plots representing differentially expressed genes (DEGs) between MCAO vs sham and SC vs MCAO groups. **(C)** Overlapping DEGs illustrated via a Venn diagram. **(D)** Heatmap showcasing hierarchical clustering of DEGs between samples. **(E, F)** GO and KEGG pathway analyses highlight significant biological processes and pathways influenced by SC in MCAO models.

Transcriptomic analysis was performed on brain tissues from the sham, MCAO, and SC-H groups. Analysis of gene expression differences revealed that in the MCAO vs sham group, there were 360 genes found to be upregulated and 282 genes found to be downregulated. Similarly, in the SC-H vs MCAO group, 384 genes were upregulated and 433 genes were downregulated (Figures 5A, B). Meanwhile, we screened out 191 differentially expressed genes (DEGs) in the three groups by a Venn diagram (Figure 5C). The heatmap was used for mapping and clustering analysis of the 40 common DEGs, including VEGFA, SPP1, FGFR1, CNKN1A, and BDNF (Figure 5D). The identified DEGs predominantly correlated with biological processes, including cell migration, proliferation, ERK cascade, and cytokine response (Figure 5E). KEGG pathway analysis indicated involvement in pathways such as the PI3K-AKT, NF-kappa B, TNF, and MAPK (Figure 5F).

### 3.6 Metabolomics analysis of the changes induced by SC

**FIGURE 6:**
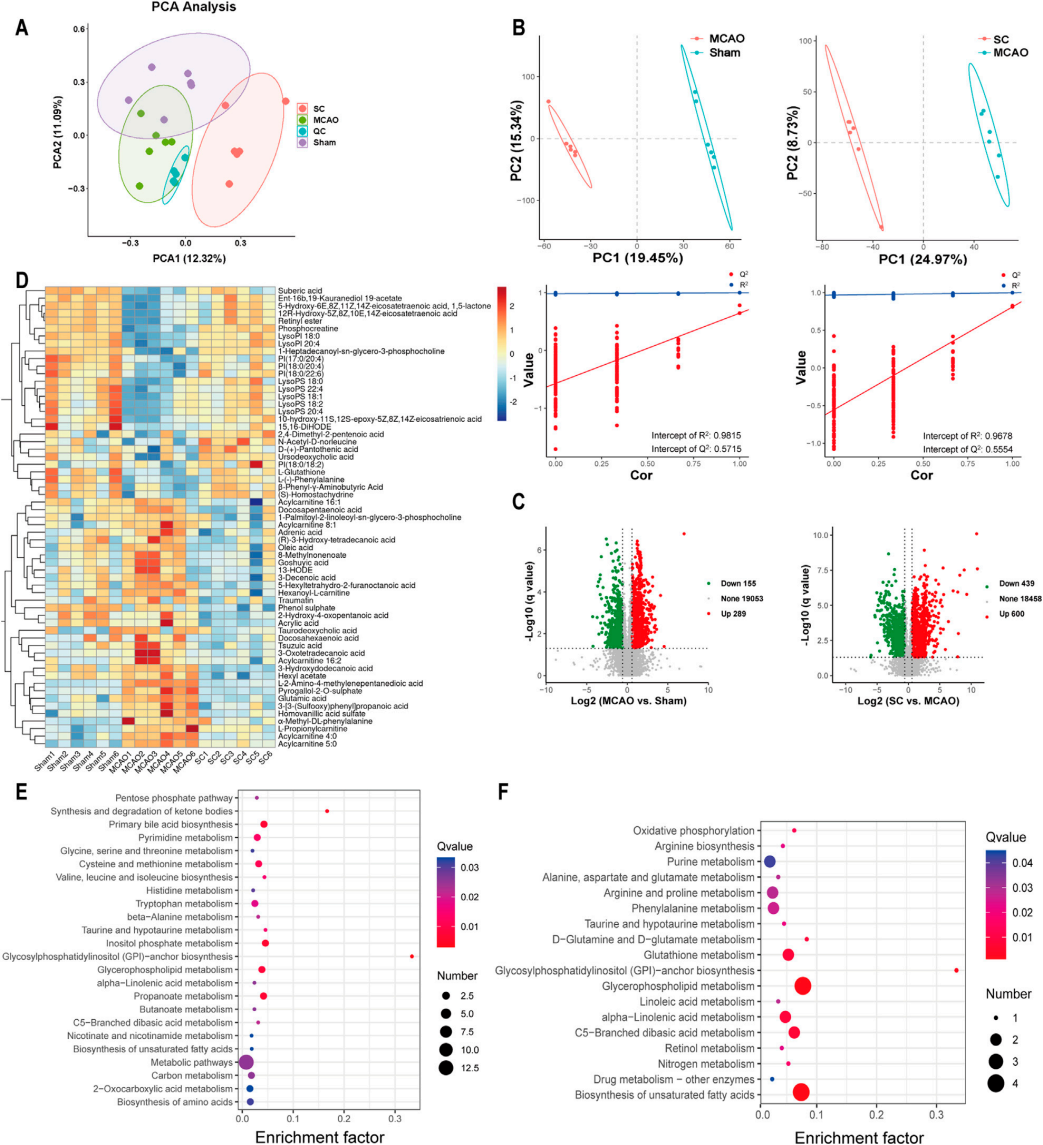
Metabolomic landscape altered by SC in ischemic stroke (n = 3). **(A)** PCA of metabolite profiles in sham, MCAO, and SC groups. **(B)** PLS-DA scores contrasting MCAO vs sham and SC vs MCAO. **(C)** Volcano plots illustrating metabolic changes between MCAO vs sham and SC vs MCAO. **(D)** Heatmap delineating key metabolites across groups. **(E, F)** Pathway analyses of significant metabolites distinguishing MCAO from sham and SC-treated groups.

Significant variations in serum metabolites among the three groups were observed through UPLC-Q-TOF-MS analysis and multivariate statistical analysis. The PCA and PLS-DA exhibited a distinct trend of separation among the three groups, indicating that the metabolites of SC differed significantly from those of the sham and MCAO groups, as presented in Figures 6A, B. A total of 444 metabolites were found to be differentially expressed in the MCAO vs sham group. In addition, 1,039 metabolites exhibited differential expression in the SC vs MCAO group, as demonstrated in Figure 6D. Sixty differential metabolites were selected and depicted in a heatmap (Figure 6C). Identification and annotation of these differential metabolites and trends in the serum of rats were shown in Supplementary Table S1. Metabolomic pathway analysis suggested associations with amino acids, glycosylphosphatidylinositol (GPI)-anchor, unsaturated fatty acids biosynthesis, alpha-linolenic acid, butanoate, glycerophospholipid, and inositol phosphate metabolism (Figures 6E, F).

### 3.7 Integrated analysis of multiple omics

**FIGURE 7:**
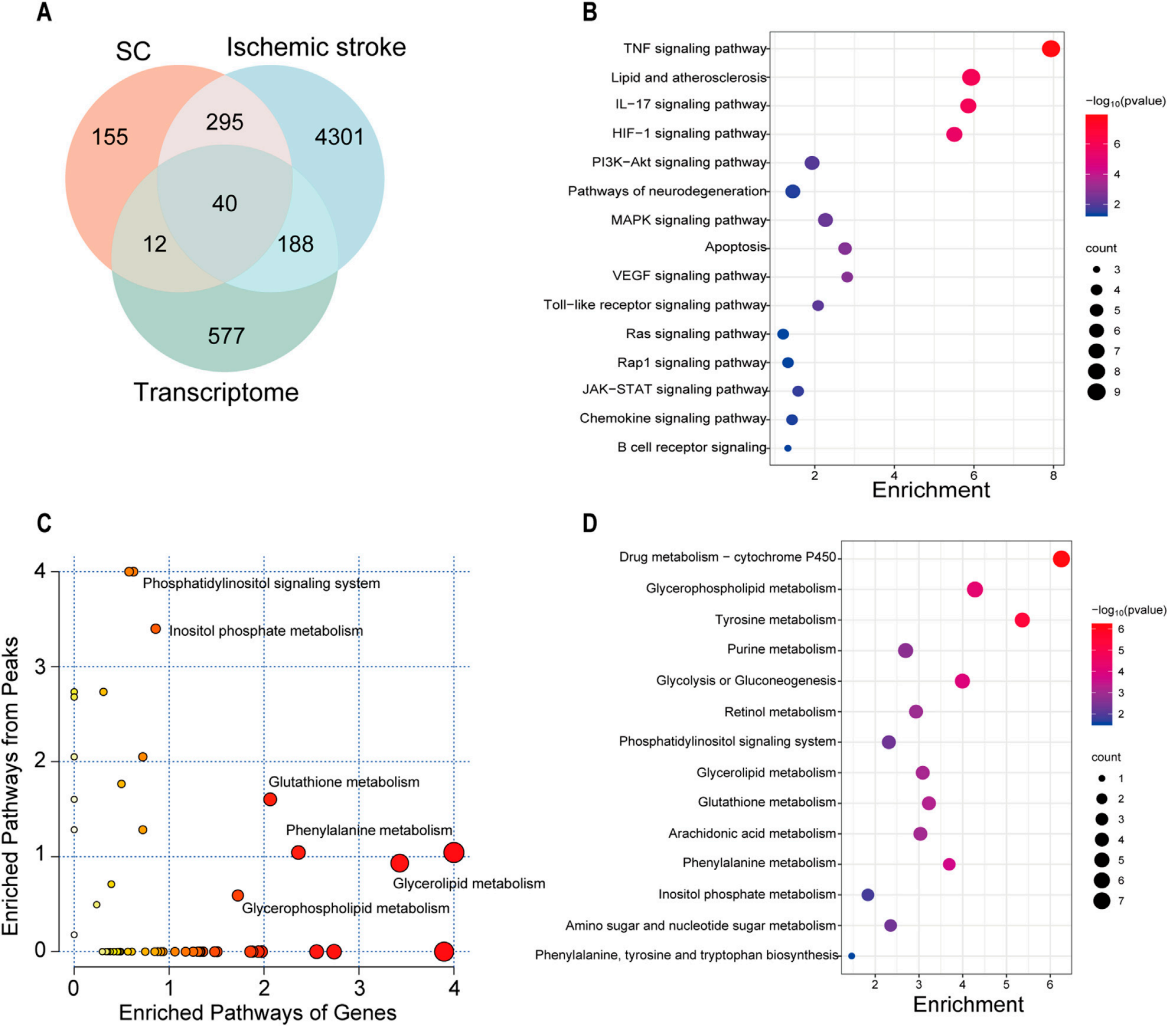
Integrated analysis of multi-omics on SC treating IS. **(A)** Venn diagram depicting the overlap of targets between SC, IS, and transcriptome. **(B)** Correlation analysis of network pharmacology and transcriptomics in the KEGG pathway. **(C)** The bubble diagram from the combined analysis of transcriptomics and untargeted metabolomics based on the MetaboAnalyst database. **(D)** Correlation analysis of untargeted metabolomics and transcriptomics in the KEGG pathway.

To obtain high-confidence candidates, we accessed 4824 IS-related genes from various databases using the keyword “Ischemic stroke” in previous studies. After comparing the predicted targets of SC, IS-related genes, and DEGs obtained from transcriptome data, with 40 intersecting targets were considered high-confidence candidates (Figure 7A), and shown in Supplementary Table S2. In the KEGG analysis, SC affected the TNF, HIF-1, VEGF, and PI3K-AKT signaling pathways, etc (Figure 7B). The correlation analysis was performed between the 817 DEGs and 1,039 metabolites using MetaboAnalyst 6.0 for Joint Pathway Analysis, and a total of 49 pathways associated with these genes or metabolites were identified. Among these metabolic pathways, including glycerophospholipid metabolism, glutathione metabolism, glycerolipid metabolism, phenylalanine metabolism, and inositol phosphate metabolism were notable (Figures 7C, D).

### 3.8 VEGFA/PI3K/AKT pathway modulation by SC

**FIGURE 8:**
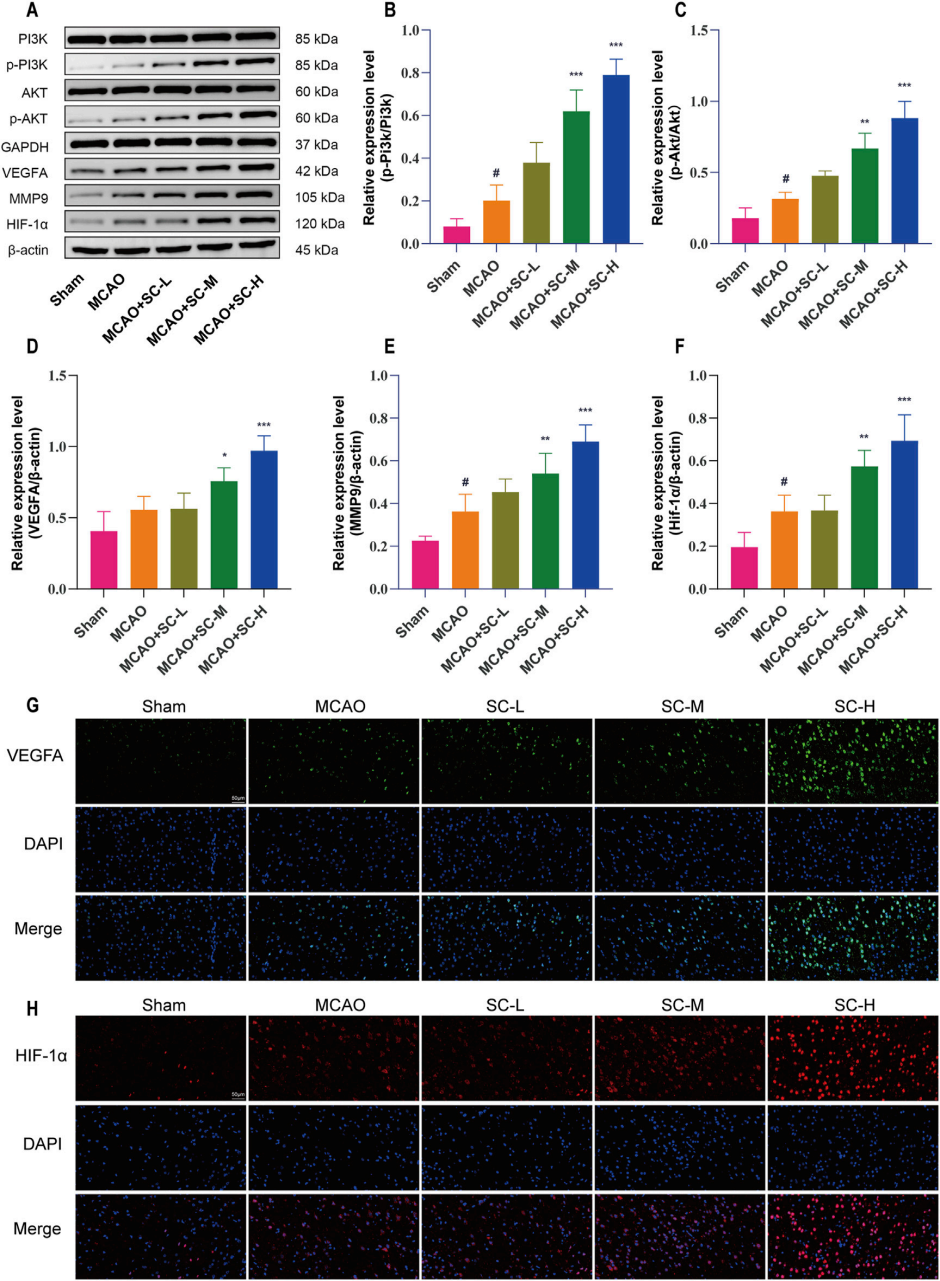
Impact of SC on key signaling proteins in the ischemic stroke pathway. **(A)** Western blot images showing expression levels of pathway proteins in ischemic brain tissue. **(B, C)** Quantitative analysis of protein expressions, specifically p-PI3K/PI3K and p-AKT/AKT ratios. **(D–F)** Quantitative evaluation of VEGFA, MMP9, and HIF-1α protein levels in brain tissues. Data presented as mean ± SD (n = 3). Statistical significance: *p < 0.05, **p < 0.01, ***p < 0.001 compared to MCAO group; #*p* < 0.05 compared to sham group. **(G, H)** Detailed immunofluorescence staining results for VEGFA and HIF-1α, respectively (Scale bar = 50 μm).

Western blot analysis indicated increases in PI3K and AKT phosphorylation levels in the MCAO group (*p* < 0.05), which were further enhanced in the SC-treated groups, with the SC-H group exhibiting the most significant increase (*p* < 0.001). Additionally, the protein expression of VEGFA, HIF-1α, and MMP9 followed a similar trend, and enhanced the VEGFA/PI3K/AKT signaling pathway by SC treatment, especially in the SC-H group (Figures 8A–F). Immunofluorescence studies, detailed in Figures 8G, H, corroborated these findings, showing an increase in VEGFA and HIF-1α expressions upon SC administration.

## 4 Discussion

Ischemic stroke is a condition characterized by high recurrence, disability, and mortality rates. Its underlying mechanisms involve multiple factors including oxidative stress, inflammation, apoptosis, and angiogenesis. In traditional Chinese medicine (TCM), herb pairs are frequently used to enhance the therapeutic effects or mitigate potential side effects in prescriptions (Wang et al., 2012; Pang et al., 2024). These herb pairs act as a bridge between single herbs and polyherbal medicines, making them a focus of research in the field of rehabilitation therapy for IS. For example, the efficacy of Danhong Injection (DHI) has been demonstrated in enhancing brain function scores and mitigating cell apoptosis in the brain tissues of individuals with IS. DHI is derived from a combination of *S. miltiorrhiza* Bunge and *Carthamus tinctorius* L. (CT) (Feng et al., 2020; Zhang et al., 2023a). Guhong injection (GHI), which is composed of CT extract and aceglutamide, has demonstrated its ability to have antiapoptotic, anti-inflammatory, and neuroprotective effects in myocardial ischemia/reperfusion injury (Zhang J. et al., 2022; Chen et al., 2023). While, because of the complexity of herbal compounds, understanding their synergistic mechanisms at the molecular level remains challenging.

Our study first confirmed that SC could have anti-cerebral ischemic injury effects, inhibit the apoptosis of neuronal cells, promote angiogenesis, and have neuroprotective effects. Based on neurobehavioral and pathological observations, treatment with mid-and high-dose SC (7.5 g/kg/d and 15 g/kg/d) for 14 days post-surgery significantly reduced neurological deficit scores, infarction area, neuronal loss, and improved neuronal morphology. We endeavored to elucidate the specific active ingredients of SC against cerebral ischemic injury. Through UPLC-Q-TOF-MS/MS analysis, we confirmed that oxypaeoniflora, benzoylpaeoniflorin, salvianolic acid B, salvianolic acid A, and tanshinone IIA had neuroprotective activity. The research found that oxypaeoniflora improves MI/R injury by activating the Sirt1/Foxo1 signaling-mediated anti-apoptotic pathway (Wang and Hu, 2020). Tanshinone IIA has been shown to have neuroprotective effects including anti-apoptotic, anti-inflammatory, and antioxidant properties (Sherawat and Mehan, 2023). In ischemic rats, salvianolic acid B has the potential to safeguard against neuronal apoptosis and mitigate neurological damage by promoting angiogenesis, a process that might be linked to the increase in VEGF and VEGFR2 through the targeting of STC1 (Bi et al., 2022). The systems pharmacology revealed that SC could regulate 67 common targets related to IS, including core targets like AKT1, TNF, PTGS2, MMP9, PIK3CA, and VEGFA, all closely linked to inflammation, cell apoptosis, and angiogenesis. The molecular docking and molecular dynamics simulation results indicated that the active ingredients mentioned above exhibited a strong affinity with core targets. This suggests that SC may have a therapeutic effect on IS by modulating these target-related pathways.

In recent years, integrated multi-omics analysis has emerged as the estimable approach to uncover the molecular mechanisms of drug action (Cheng et al., 2024; Li et al., 2024; Sun et al., 2023). We conducted a combined systems pharmacology, transcriptome, and metabolome analysis to elucidate the mechanism of action of SC against IS. Transcriptomics results revealed that SC treatment upregulated the expression of key genes, including VEGFA, secreted phosphoprotein 1 (SPP1), type I fibroblast growth factor receptor (FGFR1), cyclin-dependent kinase inhibitor 1A (CDKN1A), and brain-derived neurotrophic factor (BDNF), in pathways related to inflammation, angiogenesis, and apoptosis such as PI3K/AKT, IL-17, TNF, and NF-kappa B signaling. These findings suggest that SC’s protective effect against IS may be associated with the regulation of these pathways.

As previously reported, VEGFA is a key driver of angiogenesis and is considered indispensable in regulating blood vessel formation (Guo et al., 2021; Zhang et al., 2023b). VEGFA activates VEGF receptor-2 (VEGFR2), which, in turn, triggers downstream signaling pathways like PI3K/AKT and MEK/ERK, ultimately regulating cell development, growth, and differentiation (Kilic et al., 2006). The PI3K family of enzymes, which generates PIP3 upon activation, plays a vital role in controlling cellular proliferation and apoptosis (Koch et al., 2021; Goyal et al., 2023). The PI3K/AKT pathway has been shown to regulate angiogenesis, inflammation, and vascular endothelial homeostasis after IS (Koutsaliaris et al., 2022; Cheng et al., 2023). Additionally, PTGS2 is involved in the regulation of angiogenesis and is associated with the VEGF signaling pathway in various diseases (Jimenez-Luna et al., 2021). MMP9 is critical in degrading the basement membrane, marking the initiation of angiogenesis (Quintero-Fabián et al., 2019). HIF-1α is among the first genes to be upregulated by hypoxia in ischemic conditions and directly stimulates VEGF gene transcription by binding to the VEGF promoter (Fu et al., 2018). The integrated analysis of network pharmacology and transcriptomics revealed the VEGF pathway and PI3K/AKT signaling pathway as potential pivotal targets for SC treatment of IS. Subsequently, we investigated the potential impact of SC on the PI3K/AKT signaling pathway. Western blot and immunofluorescence staining partially validated that SC could activate the PI3K/AKT signaling pathway, induce AKT phosphorylation, inhibit degradation of HIF-1α, facilitate its accumulation, and subsequently translocate it to the nucleus for initiating transcriptional activation of VEGF. Consequently, enhanced expression of VEGF occurs which upon binding to its receptor VEGFR2 activates the downstream PI3K/AKT signaling cascade. These findings underscore the remarkable potential of SC in promoting recovery from IS and safeguarding neural tissue. There are still limitations in our study and more experiments are needed to explore the intrinsic mechanisms.

In addition, the transcriptomics analysis revealed that a substantial proportion of the genes displaying differential expression were significantly enriched in metabolic pathways. This observation highlights the potential impact of SC on regulating metabolic processes. Clinical studies have associated higher blood and cerebrospinal fluid concentrations of glutamate with neurological deficits in acute ischemic stroke (Castillo et al., 1997). Furthermore, branched-chain amino acids like valine, leucine, and isoleucine have been linked to various stages of stroke and may serve as diagnostic markers for IS (Sidorov et al., 2019). Altered levels of lyso-PI and lyso-PS in serum are indicative of impaired phospholipid metabolism and could potentially serve as biomarkers for IS (Sun et al., 2017). Acetylcarnitine, linked to fatty acid metabolism, increased during IS and is considered a potential target for IS therapy (Huang et al., 2023). Integrated analysis of metabolomics and transcriptomics revealed that SC treatment corrected disturbances in amino acids, glycerophospholipids, sphingomyelin, and fatty acid metabolism in MCAO rats. Glutamate, phenylalanine, leucine, lyso-phosphatidylinositol (lyso-PI), lyso-phosphatidylserine (lyso-PS), and acylcarnitine are key metabolites in the neuroprotective effect of SC.

One limitation of this study is the exclusive use of male rat models, which overlooks the physiological and hormonal disparities between males and females. It is crucial to acknowledge that the therapeutic effects of the SC herbal combination may vary based on sex. To obtain a more comprehensive understanding of its efficacy and safety, future studies should incorporate female rats as well. By including both sexes in the research, more generalized conclusions can be drawn, better reflecting the potential clinical applications of the SC herbal combination across diverse patient populations.

## 5 Conclusion

In summary, our research first time demonstrates the neuroprotective efficacy of SC in a rat MCAO model, mediated through the activation of the VEGFA/PI3K/AKT pathway, as evidenced by multi-omics analyses. These results suggest SC’s potential as a therapeutic agent for IS. It is worth mentioning that the scope of this investigation is restricted to research conducted at the animal level. Subsequently, additional exploration is required to assess the clinical potential of SC.

## Data Availability

The transcriptome library construction and sequencing data presented in the study was deposited in the NCBI SRA BioProject repository, and the accession number was PRJNA1110725.

## Abbreviations

IS, Ischemic stroke; SM, Salvia miltiorrhiza; CM, Cortex moutan; SC, Salvia miltiorrhiza-Cortex moutan; MCAO, middle cerebral artery occlusion; STRING, String online platform; C-T-P, compound-target-pathway; SPP1, secreted phosphoprotein 1; FGFR1, type I fibroblast growth factor receptor.
