# Supplementary figures and images for: Combining systems pharmacology, metabolomics, and transcriptomics to reveal the mechanism of Salvia miltiorrhiza-Cortex moutan herb pair for the treatment of ischemic stroke
Source: Front Pharmacol. 2024 Sep 9;15:1431692. doi: 10.3389/fphar.2024.1431692 (PMC11417465; doi:10.3389/fphar.2024.1431692)

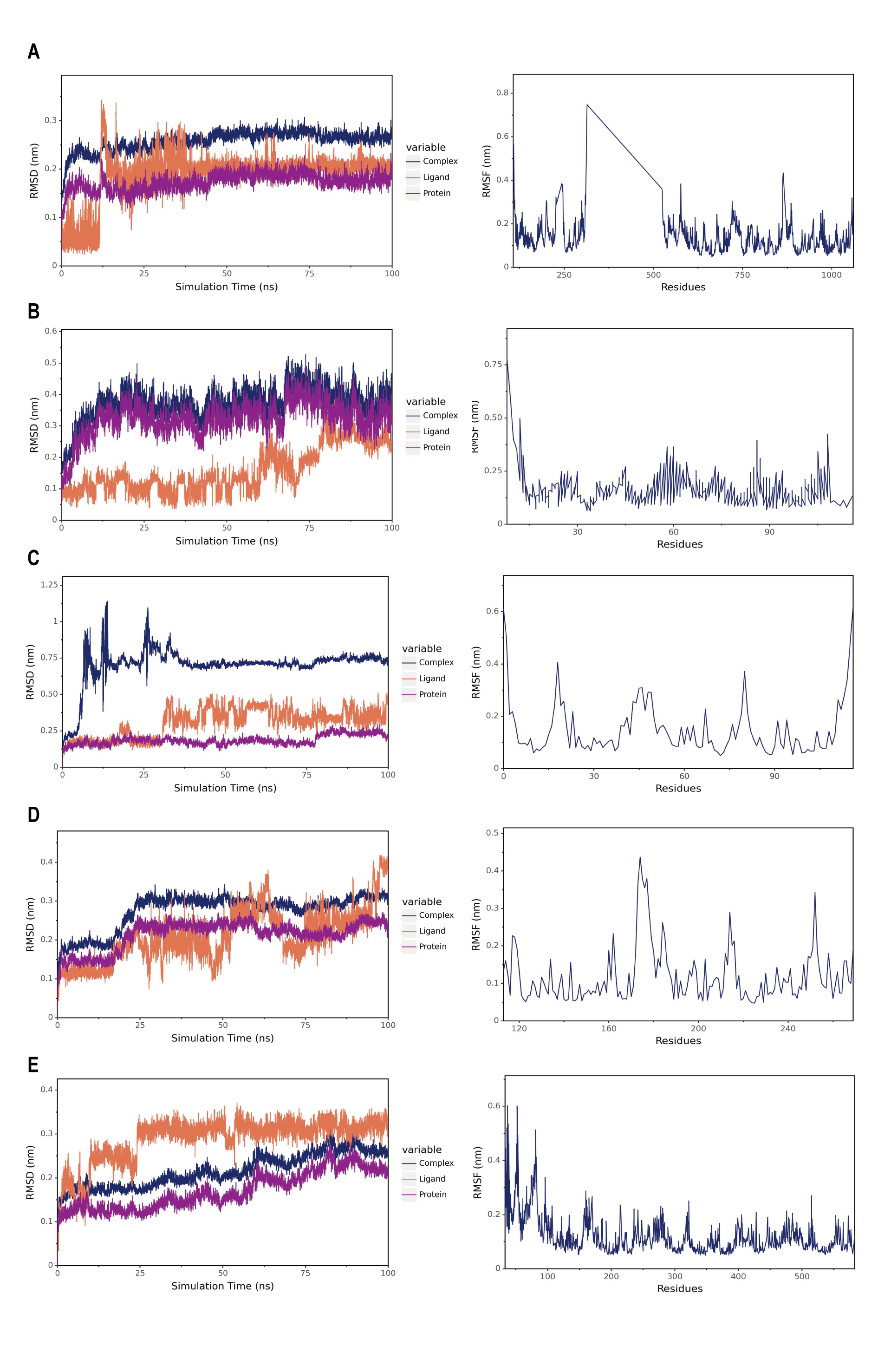

Supplement: Supplementary file 3 [file Image1.TIF]
